# Transition of pediatric patients with bronchiectasis to adult medical care in the Northern Territory: A retrospective chart audit

**DOI:** 10.3389/fped.2023.1184303

**Published:** 2023-04-28

**Authors:** Kobi L. Schutz, Nicholas Fancourt, Anne B. Chang, Peter Morris, Rachel Buckley, Edwina Biancardi, Kathryn Roberts, James Cush, Subash Heraganahally, Gabrielle B. McCallum

**Affiliations:** ^1^ Child Health Division, Menzies School of Health Research, Charles Darwin University, Darwin, NT, Australia; ^2^ School of Nursing, Charles Darwin University, Darwin, NT, Australia; ^3^ Department of Respiratory and Sleep Medicine, Queensland Children’s Hospital Queensland University of Technology, Brisbane, QLD, Australia; ^4^ Global and Tropical Health Division, Menzies School of Health Research, Charles Darwin University, Darwin, NT, Australia; ^5^ Department of Respiratory and Sleep Medicine, Royal Darwin Hospital, Darwin, NT, Australia; ^6^ Department of Paediatrics, Royal Darwin Hospital, Darwin, NT, Australia; ^7^ College of Medicine and Public Health, Flinders University, Adelaide, SA, Australia

**Keywords:** bronchiectasis, transition plan, transition, adult care, pediatric care

## Abstract

**Background:**

Bronchiectasis is increasingly being recognized to exist in all settings with a high burden of disease seen in First Nations populations. With increasing numbers of pediatric patients with chronic illnesses surviving into adulthood, there is more awareness on examining the transition from pediatric to adult medical care services. We undertook a retrospective medical chart audit to describe what processes, timeframes, and supports were in place for the transition of young people (≥14 years) with bronchiectasis from pediatric to adult services in the Northern Territory (NT), Australia.

**Methods:**

Participants were identified from a larger prospective study of children investigated for bronchiectasis at the Royal Darwin Hospital, NT, from 2007 to 2022. Young people were included if they were aged ≥14 years on October 1, 2022, with a radiological diagnosis of bronchiectasis on high-resolution computed tomography scan. Electronic and paper-based hospital medical records and electronic records from NT government health clinics and, where possible, general practitioner and other medical service attendance were reviewed. We recorded any written evidence of transition planning and hospital engagement from age ≥14 to 20 years.

**Results:**

One hundred and two participants were included, 53% were males, and most were First Nations people (95%) and lived in a remote location (90.2%). Nine (8.8%) participants had some form of documented evidence of transition planning or discharge from pediatric services. Twenty-six participants had turned 18 years, yet there was no evidence in the medical records of any young person attending an adult respiratory clinic at the Royal Darwin Hospital or being seen by the adult outreach respiratory clinic.

**Conclusion:**

This study demonstrates an important gap in the documentation of delivery of care, and the need to develop an evidence-based transition framework for the transition of young people with bronchiectasis from pediatric to adult medical care services in the NT.

## 1. Introduction

### 1.1. Why transition of adolescents with bronchiectasis to adult care is important?

While once considered rare, the awareness of bronchiectasis among children and adolescents in the global community has increased over the last several decades (1). Bronchiectasis is now recognized in all settings, although more common and severe in First Nations people (2, 3). To date, reliable prevalence data for childhood bronchiectasis is lacking; however, current global estimates range from 0.2 to 735 cases per 100,000 children (2, 4). The highest documented burden, however, is reported among socially disadvantaged people of high-income countries, such as Australian First Nations children (1 in 68) (5) and those in low- and middle-income countries (4).

Bronchiectasis is a major contributor to chronic lung morbidity (1) and mortality particularly in Australian First Nations people (4, 6); however, it remains one of the most neglected diseases in respiratory health globally (7). Importantly, if management is suboptimal, this may be associated with poorer clinical outcomes (8) and premature death in the third and fourth decades of life (8). A striking example is among First Nations adults from Central Australia in the Northern Territory (NT) where the mortality gap is >20 years compared to other Australian adults with bronchiectasis (8, 9). Another paper recently reported the poorer outcomes between First Nations and non-First Nations adults with bronchiectasis in the NT further highlighting the disparities (10); thus, there is a need for prompt interventions to reduce this inequity. To improve clinical outcomes and quality of life for people with bronchiectasis, optimizing evidenced-based management that is across the life course, including transition of care (11), is needed.

There are, however, few management guidelines available for bronchiectasis in the current era. These include the Thoracic Society of Australia and New Zealand (TSANZ) pediatric and adult bronchiectasis guidelines that were published in 2002 (12) with updates in 2008 (13), 2010 (14), 2015 (15), and 2023 (16); the British Thoracic Society guidelines published in 2010, although are only specific to adults (17); and, most recently, the European Respiratory Society (ERS) that published the first international evidence guidelines on pediatric bronchiectasis (1). These guidelines share many of the same principles as the TSANZ, noting however some differences. For example, the ERS guidelines recommend children are reviewed every 3–6 months (1) to monitor lung function, general health, and wellbeing, whereas TSANZ recommends monitoring every 6 months (15). Current recommendations for adult patients, on the other hand, include an annual review with a multidisciplinary team (15).

A key aspect to optimizing bronchiectasis management across the life course includes the process of transitioning patients from pediatric to adult services. The transition process for adolescents (from here respectfully termed “young people” and who are aged ≥14 years) with chronic conditions was first described in the 1980s as the “purposeful, planned movement of adolescents and young adults with chronic physical and medical conditions from child-centered to adult-orientated health care systems” (18). An ideal transition framework provides care that is age appropriate and coordinated (18, 19), involving ongoing communication between all parties over a period of time, and the sharing of information between child health practitioners, patients, caregivers, and adult practitioners (19).

Historically, the need for transition from pediatric to adult care for chronic conditions was not considered a high priority due to low survival rate beyond 21 years for some chronic illnesses (18). However, with increasing survival in the modern era, a greater number of patients with chronic illnesses are transitioning into adult care, and thus there is greater importance on the transition process to improve longer-term outcomes (20). Poor transition, however, can leave patients and their caregivers without the right tools to manage individualized care appropriately (21). Furthermore, qualitative research has found that when the transition of young people is uncoordinated, patients often struggle to adjust to the jump in expectation and level of independence required for adult services (22). On the other hand, well-coordinated transition can support young people to develop autonomy in their care, engage with healthcare providers, reduce risk taking behaviors, and improve social and emotional wellbeing (20).

In several chronic diseases, such as cystic fibrosis (CF), diabetes, and congenital heart disease, improved health outcomes have been observed when transition processes are used (23). Furthermore, the use of disease-specific transition frameworks from pediatric to adult services have shown to improve the autonomy of individualized care and effective engagement with care providers (23). However, currently none are available for young people with bronchiectasis or in at-risk populations (24), such as Australian First Nations people.

Recently, a small New Zealand study described the inequitable care young people with bronchiectasis received post-transition from pediatric to adult services compared to those with CF (24). Despite the bronchiectasis cohort having more severe disease, they received less preparation for transition and post-transition care compared to the CF cohort who had improved transition success, with fewer losses to follow up in the health system, and were 20.1 times more likely to attend scheduled post-transition appointments (*p* < 0.0001) than the bronchiectasis cohort (24).

The data above suggest that there are major disparities between specific respiratory diseases, an absence of a transition framework, and a need to improve services and clinical outcomes for young people with bronchiectasis. The importance of this issue in our region is currently not known. Thus, we undertook a retrospective medical chart audit to describe what processes, timeframes, and supports were in place to transition young people with bronchiectasis from pediatric to adult services in the NT from 2007 to 2022 to define steps for improvement and to determine outcomes, at 12 and 24 months post-transition.

## 2. Methods

### 2.1. Study participants and setting

Study participants were identified from a larger prospective study conducted at the Royal Darwin Hospital (RDH), NT, Australia, which recruited children from 2007 to 2022. The aforementioned study enrolled children aged <18 years undergoing bronchoscopy and/or high-resolution computed tomography (HRCT) for clinical reasons (e.g., chronic or recurrent wet cough) (25). Only young people aged ≥14 years on October 1, 2022, with a radiological diagnosis of bronchiectasis on HRCT (as determined by the pediatric respiratory specialist) were eligible for this current study.

The local Human Research Ethics Committee of the NT Department of Health and Menzies School of Health approved this current study (HREC 2022-4325) and provided a waiver of consent for this current analysis, as primary caregivers from the original study had provided written informed consent (HREC 06/73). The Menzies Australian First Nations Reference Group for Child Health also provided their support for this study.

### 2.2. Data collection

Demographics, medical history, and details of the primary bronchiectasis diagnosis [e.g., date of HRCT scan, etiology, lobar involvement, previous respiratory hospitalizations, severity scoring using (Bhalla: range 0–25 (26) and modified Bhalla: range 0–48) (27), with a higher reflecting greater severity) (25, 28) were extracted from the original study database and entered into a REDCap database (29, 30).

The medical records were then reviewed for evidence of any documented transition planning from age ≥14 to 20 years that included referral letters, booked and attended outpatient appointments, attendance at adult respiratory appointments, and hospital admission or emergency department (ED) presentations for respiratory exacerbations. In addition, the electronic medical records of NT Government (NTG) serviced remote health clinics were reviewed using the Primary Care Information system (PCIS). PCIS entries were reviewed for booked and attended health appointments with health professionals for evidence of documented transition planning as listed above.

### 2.3. Analysis

Summary statistics were presented as median and interquartile range (IQR: 25%–75%) for continuous data (dependent of data distribution) and frequency (percentage) for categorical data. Data from the original bronchiectasis study was imported into REDCap and data for this current study were entered directly into REDCap (29, 30). We analyzed the data using Stata version 17 (Stata Corp College Station, TX, United States).

## 3. Results

### 3.1. Baseline participant characteristics

A total of 102 young people were eligible to transition from pediatric to adult services between 2007 and 2022 (Table 1). The median age at the time of bronchiectasis diagnosis was 3.94 years (IQR: 2.03–6.15); 53 (52%) were males and 97 (95%) were identified as First Nations people. Most of the First Nations cohort (92%) resided in a remote community of the NT (>100 km from a tertiary hospital), with 61% having their healthcare serviced by NTG Health Clinics. Post-infection was the most common etiology (94%) with 87 (85.3%) having ≥3 lobes involved.

**Table 1:** Baseline characteristics at time of bronchiectasis diagnosis.

Variable	*n* = 102 (%)
Demographics	
Age (years)^a^	3.9 (2.0–6.1)
Male sex	53 (52)
Ethnicity	
First Nations	97 (95)
Non-First Nations	5 (5)
Low birthweight, <2.5 kg	39 (38.2)
Preterm, <37 weeks	34 (33.3)
Remote, >100 km from RDH	92 (90.2)
Number of lobes affected	
1	4 (3.9)
2	11 (10.8)
3	27 (26.5)
4	27 (26.5)
5	26 (25.5)
6	7 (6.9)
Etiology	
Post-infection	96 (94.1)
Chronic neonatal lung disease	2 (2)
Others^b^	4 (3.9)
Severity	
Respiratory hospitalizations (ever)	2 (1–4)
Respiratory hospitalizations (last 12 months)	0 (0–2)
Bhalla score^a^^,^^c^	4/97 (4–8)
Modified Bhalla score^a^^,^^d^	8/97 (6–10)

RDH, Royal Darwin Hospital.

Note: Only *n* = 97 high-resolution computed tomography scans were available for Bhalla and modified Bhalla scoring.

^a^ Data are presented as *n* (%) or median (25th–75 percentile), unless otherwise indicated.

^b^ Foreign body, *n* = 2; specific antibody deficiency, *n* = 1; hypogammaglobulinemia, *n* = 1.

^c^ Bhalla (range 0–25).

^d^ Modified Bhalla (range 0–48).

### 3.2. Interactions with medical services, respiratory-related illnesses, and evidence of transition (≥14 to <18 years)

At the conclusion of the observation period (October 1, 2022), the median age was 16 years (IQR: 15–17.8), with 76 (74%) young people still being under 18 years. In the last 2 years of the observation period, five young people had admissions to hospital for exacerbation of their bronchiectasis, with two young people having greater than two presentations.

Overall, only 12/102 (12%) young people aged between ≥14 to <18 years had evidence of a pediatric respiratory specialist outpatient booking made at the RDH, which is the main tertiary hospital for the Top End of the NT. Of the 12, one had one appointment booked but did not attend and was not rebooked, one had two appointments booked and attended one, and one had six appointments booked and attended one. Three had two bookings and attended both, four had one booking and attended all, and two had >3 appointments booked per year and attended all.

Of the 92 young people who lived in a remote setting, there was evidence of 43 (46.7%) being placed on a recall list for community pediatric follow-up. Of the 43, there were 141 separate booked appointments with either a general pediatrician or a trainee, district medical officer (DMO), or other specialists. Of the booked appointments, 35 (24.8%) were for other specialist areas such as cardiology, allergy, and ears, nose, and throat follow-up of which 14 (40%) of these appointments were not attended. Of the 106 general pediatric appointments made, only 59 (50.3%) were attended. These included three relating to diabetes, two general health checks, and one guardianship meeting. Another three had attendance details recorded but no medical record was available. Of the 106 general pediatric appointments made, 9 (8.5%) mentioned transition or discharge and 41 (39%) had some discussion regarding the current state of the young person's respiratory health. None of these appointments mentioned spirometry, with only one receiving a chest x-ray over this time.

Of the 10 urban young people (lived within 100 km of a tertiary hospital), 5 (50%) had booked appointments in the pediatric outpatient department at RDH. There were 48 separate appointments made, 18 (37.5%) of these were for other specialist areas including 10 endocrine, 7 cardiac, and 1 dietician appointment. There were 7 general pediatric and 23 respiratory-specific appointments made. All respiratory appointments were attended with 22/23 (95.7%) having spirometry documented. Among the urban cohort, evidence of transition or discharge was documented only for two young people.

Regarding interactions with the ED, 34 (33.3%) young people presented for 96 separate presentations. Of these, 78 (81.2%) were for wound management, abdominal concerns, acute rheumatic fever, fever, antenatal, dental, and mental health concerns, with the remaining 18 (18.8%) being respiratory-related. Of these, seven presentations were for exacerbations requiring admission to one of the pediatrics wards, and seven were discharged home. Chest x-rays were performed on three participants, two of whom were admitted to hospital and one who was discharged home. There was, however, no documented evidence of spirometry being performed. The remaining four presentations were booked admissions for bronchiectasis tune up with intravenous antibiotics that were admitted *via* the ED.

Overall, there was limited evidence of transition planning among the 102 young people, with only 9/102 (8.8%) having documentation of transition planning or actual discharge from the pediatric service. Of these eight, the process of transition was discussed twice with two young people, with the first discussion approximately 1 year prior to discharge. Only one of these, however, had mention of a formal handover to adult services where an appointment was made. The medical records of the other six indicated that the young adults had been discharged from routine pediatric follow-up with three recommending follow-up with general practitioners for exacerbations. The other three young people had no documentation of future planning.

### 3.3. Health engagement (≥18 to <20 years)

There were 26/102 (25.4%) participants who had turned 18 prior to October 1, 2022, with 25 (96%) living in a remote area. There was no evidence in the medical records of any young person attending either a dedicated adult respiratory clinic at RDH or adult outreach respiratory clinic held in over 20 communities across the NT. There was evidence that two attended adult clinics for hematology and ears nose and throat, and 13 adults presented to ED collectively 25 times. Only two of these presentations were respiratory-related, with one being discharged home and the other admitted to hospital. In both, there was no documentation of spirometry being performed.

## 4. Discussion

In this study involving 102 participants, we sought to describe the transition process of young people with bronchiectasis from pediatric to adult services in the NT from 2007 to 2022. We showed that transition processes are poorly documented in this cohort, with less than 9% of participants aged ≥14 to <18 having evidence of transition planning in either a tertiary or remote primary care setting, and of the 26 young people who were eligible to transition, there was no evidence of attendance at specialist adult respiratory clinics at RDH or adult outreach respiratory clinics.

Importantly, our findings are not dissimilar to other reviews of transition processes for young people with bronchiectasis. For example, a retrospective study in Alaska on adult outcomes of childhood bronchiectasis recently reported that among 29 patients who transitioned to adult care, 62% had no mention of bronchiectasis in their medical documentation at or after 20 years of age despite 72% remaining symptomatic (31). Having no record of bronchiectasis history resulted in inadequate management of exacerbations or new investigations for presumed adult-onset bronchiectasis (31).

Reasons for the apparently low transition rate can be speculated. The management of bronchiectasis is chronically underfunded with clear inequity compared to other respiratory disorders, even in tertiary hospitals. In addition to the Alaskan data described above (31), a Sydney study found that children with bronchiectasis received less care (medical and physiotherapy reviews) and had significantly poorer lung function compared to age-matched children with CF (32). Furthermore, in our setting at RDH, the disparity between chronic respiratory diseases and other chronic disease is further exacerbated, with no dedicated chest physiotherapy services in spite of the large burden of disease (25). Globally, it is known that although chronic respiratory diseases are a leading cause of death and disability worldwide, they receive proportionately less research funding and public attention than other diseases (33). It is important to also recognize that 90% of participants in this study lived in remote NT communities. In these communities, the turnover of staff is 148% per annum for registered nurses and 80% for Aboriginal Health Practitioners (34), increasing the risk of potentially reducing continuity of care for this vulnerable population (34).

In addition, in line with our efforts over the decades, as bronchiectasis is diagnosed earlier in these children, the disease severity (defined by Bhalla scores) at the time of diagnosis was relatively low. While we could not define disease severity at the time of transition, the low radiological disease severity in our cohort may partially account for our low transition rates. Other possible reasons for low transition include resolution and/or improvement of the disease. If bronchiectasis is appropriately treated, early resolution/improvement rates may account for up to 64% in children and young people (35, 36). Symptom improvement has been noted in adolescent cohorts since the 1960s–1970s, where a period of improved wellness and reduced exacerbations when compared to childhood is observed, often until the third and fourth decades of life (37, 38). Recent work among Alaskan Native, New Zealand, and Australian young people with chronic suppurative lung disease/bronchiectasis further support these data, which showed a decrease in the annual acute lower respiratory infections with increasing age (39, 40). It is possible but remains unknown that during this wellness period, a number of patients may be discharged from pediatric clinics on the account of being clinically well, with no transition to adult care being offered.

Our study has several limitations that included the retrospective nature, incomplete medical records, and inconsistencies in record keeping between medical practitioners. Due to the large number of remote participants in this study, most were not treated by respiratory physicians. Their geographical isolation meant that they were usually managed in their local communities by DMOs or visiting pediatricians. We were also not able to access local general practitioner records or non-government health service providers for all participants for evidence of health engagement. We could not capture every young person with bronchiectasis as we only included young people enrolled in the preceding (HREC 06/73) study, so we may have missed some cases of successful transition.

Nevertheless, recommendations from this study include the urgent need to develop a bronchiectasis transitional framework using evidence-based guidelines (1, 16) and quality standards (40). We recommend involving both Government and Aboriginal Controlled Medical Services in a round table discussion, alongside healthcare practitioners involved in the care of patients with bronchiectasis across the lifespan, to inform the development of this framework for the NT. This should support the successful transition from pediatric to adult medical care. However, there is still clinical uncertainty around which children will benefit from review by specialists outside their community, and the specific management goals of the local primary healthcare team when bronchiectasis is asymptomatic. It is thus imperative that the transition pathway is both inclusive and flexible enough to meet the complex needs among this high-risk population. Focus groups involving young people and their caregivers from urban and remote communities would also help establish the priorities of ongoing care from the patient's perspective.

## 5. Conclusion

Previous studies have shown that disease-specific comprehensive transition frameworks can improve health-related outcomes. Currently, there is a lack of transition recommendations in relation to bronchiectasis, with none in at-risk First Nations young people. Despite the high burden of bronchiectasis among First Nations people in the NT, the proportion of young people with a documented transition plan remains low. This highlights the need for a structured transition framework to support the transition from pediatric to adult medical care to ensure the continuation of health engagement to improve long-term health outcomes.

## Data Availability

The datasets presented in this article are not readily available because as per our institutions’ policies involving Australian First Nations children and in accordance with national guidelines, we are unable to share individual participant data as specific consent for this was not obtained. Requests to access the datasets should be directed to the corresponding author.
